# Overview of the gene regulation network and the bacteria biotope tasks in BioNLP'13 shared task

**DOI:** 10.1186/1471-2105-16-S10-S1

**Published:** 2015-07-13

**Authors:** Robert Bossy, Wiktoria Golik, Zorana Ratkovic, Dialekti Valsamou, Philippe Bessières, Claire Nédellec

**Affiliations:** 1 Unité Mathématique, Informatique et Génome MIG INRA UR1077 - F-78352 Jouy-en-Josas - France; 2 LaTTiCe UMR 8094 CNRS, 1 Rue Maurice Arnoux, F-92120 Montrouge - France; 3 LIMSI UPR3251 CNRS, BP 133, F-91403 Orsay - France

## Abstract

**Background:**

We present the two Bacteria Track tasks of BioNLP 2013 Shared Task (ST): *Gene Regulation Network *(GRN) and *Bacteria Biotope *(BB). These tasks were previously introduced in the 2011 BioNLP-ST Bacteria Track as *Bacteria Gene Interaction *(BI) and *Bacteria Biotope *(BB). The Bacteria Track was motivated by a need to develop specific BioNLP tools for fine-grained event extraction in bacteria biology. The 2013 tasks expand on the 2011 version by better addressing the biological knowledge modeling needs. New evaluation metrics were designed for the new goals. Moving beyond a list of gene interactions, the goal of the GRN task is to build a gene regulation network from the extracted gene interactions. BB'13 is dedicated to the extraction of bacteria biotopes, *i.e*. bacterial environmental information, as was BB'11. BB'13 extends the typology of BB'11 to a large diversity of biotopes, as defined by the OntoBiotope ontology. The detection of entities and events is tackled by distinct subtasks in order to measure the progress achieved by the participant systems since 2011.

**Results:**

This paper details the corpus preparations and the evaluation metrics, as well as summarizing and discussing the participant results. Five groups participated in each of the two tasks. The high diversity of the participant methods reflects the dynamism of the BioNLP research community.

The highest scores for the GRN and BB'13 tasks are similar to those obtained by the participants in 2011, despite of the increase in difficulty. The high density of events in short text segments (multi-event extraction) was a difficult issue for the participating systems for both tasks. The analysis of the BB'13 results also shows that co-reference resolution and entity boundary detection remain major hindrances.

**Conclusion:**

The evaluation results suggest new research directions for the improvement and development of Information Extraction for molecular and environmental biology. The Bacteria Track tasks remain publicly open; the BioNLP-ST website provides an online evaluation service, the reference corpora and the evaluation tools.

## Background

### Motivation and related work

Large-scale experimental approaches in the field of biology shift the focus of researchers towards transversal questions that involve very diverse biological knowledge. The researcher needs new tools to deal with the growing number of relevant publications. The domain of text-mining for biology (BioNLP) develops automatic methods to assist the analysis of knowledge expressed in natural language scientific articles. Periodic shared tasks measure the progress of the community methods by formally comparing the method predictions to a reference annotation on test data [1,2]. The goals of the shared tasks evolve with the advances in BioNLP, moving towards a better adaptation to the needs of biologists. It is reflected through the diversity of biology questions (*e.g*. regulation, disease, metabolism, and environment), types of documents (*e.g*. abstracts, papers, Web pages) and the related biologist research activity (*e.g*. knowledge curation, system modeling, and data normalization).

The third series of BioNLP Shared Task that took place in 2013 (BioNLP-ST'13) proposed six tasks under the *Knowledge Base *domain. BioNLP-ST'13 encourages the development of methods that improve the extraction of fine-grained complex events in systematic and concise ways [3]. The common organization of BioNLP-ST'13 includes an official evaluation of the participant systems by an automatic comparison of their predictions on the test sets to reference data. The evaluation took place at a fixed date after a period for the training of methods using the reference corpora provided by the task organizers. The two Bacteria Track tasks were organized within this framework.

The creation of the BioNLP Bacteria Track in 2011 [4] followed the LLL initiative in 2005 [5]. It was motivated by research questions brought up by bacteria research that encompass all levels of knowledge from the molecular to the physiological and phenotypic levels. This is exemplified by the GRN and BB tasks.

### Gene Regulation Network

Gene regulation networks are key components in the understanding of cell processes and more generally of living organisms. More and more research efforts are being invested in the design of regulation networks for species of all kingdoms [6,7]. Systems biology aims to integrate the knowledge from heterogeneous sources in consistent predictive models of gene regulation [8,9]. Beyond experimental data, which is the main source to date, the abundance of regulation descriptions in literature has been a strong motivation for the development of dedicated Information Extraction (IE) methods [5,10,11]. The goal of the LLL task was the extraction of binary directed interactions between the agent protein and the target gene from which the regulation network can be derived in a straightforward way. The gene interaction information extraction task has posed a challenge for years due to the spread of information over large collections of scientific data, the complexity of the underlying biological phenomena and the linguistic diversity of the descriptions.

[2] and [12] showed that the use of a fine-grained biological model for the representation of the events facilitates the understanding and validation of the extracted knowledge by the biologists and its integration with other data sources. A fine-grained model was implemented in the *Bacteria Gene Interaction *(BI) task in 2011. It describes in detail interactions at the biological level and underlying cellular mechanisms at the molecular level [4]. The activation of the transcription of a gene is an example of a biological interaction. The physical binding of a protein to a DNA site is an example of a molecular level phenomenon.

The BI model is formalized by biological entities and n-ary events between entities and events. The biological entities are mainly proteins and genes, their subparts (*e.g*. site), families (*e.g*. gene cluster) and aggregates (*e.g*. protein complex).

The GRN task goes one step beyond BI by making the design of the regulation network its primary goal. It has several benefits compared to the BI text-bound event extraction. Regulation networks are needed by biologists in order to enrich their biological models and to integrate text knowledge with other sources of knowledge. Because the knowledge extracted from text is directly bound to its sources, end-users can more easily assess its quality, compared to other bioinformatics methods such as transcriptomic profile screening [13]. Moreover, the evaluation of regulation network quality better reflects the biologist needs because it abstracts from text-mining peculiarities, such as the linguistic complexity of the text descriptions and information redundancy.

The GRN annotation model is built upon the BI model, and also includes inference rules to automatically deduce the regulation network from text-bound events. The inference rules provides a continuity between event-based extractions and the regulation network, allowing to benefit from both types of knowledge.

The biological question behind the LLL and BI tasks is the cell process regulation network of the model bacterium *Bacillus subtilis *(*Bs*) with a focus on sporulation, one of the most studied developmental processes. This choice was motivated by the abundance of publicly available information in PubMed abstracts and the richness of the biological phenomena described in them.

### Bacteria Biotope

The previous work on the Bacteria Biotope task (BB'11) has stressed the importance of microorganism environment information. The formal description of biotopes and their properties is an essential step for the study of interactions between the organisms and their environment. In particular, it is needed in order to correlate genetic specificity to environmental properties and to explain the adaptation of organisms to their habitats and their evolution. The application domains of this fundamental research are broad, from the health of humans, plants, and animals, to food processes including plant growth enhancement [4]. Biotope descriptions are abundant in scientific documents, but they cannot be used as such for biological studies. Their form is extremely variable: the biotope descriptions may be very complex from a linguistic point of view, including many embedded biotope names and properties. A normalization of biotope descriptions using a reference is required for their comparison. The extraction of the relations between the organism and the biotope entities is also difficult to automate due to the abundance of entity mentions in short spans of texts. This motivated the organization of the Bacteria Biotope Task in 2011. The goal of BB'11 was the identification and categorization of bacterial habitat entities in natural language texts, their linking to their subparts (*i.e*. part-of event) and their linking to the bacteria that live there (*i.e*. localization event). The good results of the participants on BB'11 demonstrate the feasibility of this IE task.

Since 2011, the development of new sequencing techniques has had a major impact on the field of metagenomics. Metagenomics studies microorganism sequences in their environments, thus avoiding strain cultivation. The number of metagenomics studies has grown exponentially in the last few years. This has resulted in a considerable increase in the diversity of the microorganisms that can be studied. This is appealing for information extraction tools that can automatically analyze biotope descriptions of the microorganisms so that these biotopes and genes from different metagenomics experiments can be compared on a large scale.

The categorization of biotopes is a form of normalization that is necessary for the generalization of biotope observations. BB'11 defined seven broad biotope categories that were *a priori *considered as relevant for biological studies. Participant methods had to categorize the extracted biotope mentions according to these categories. The limited number of categories affects the ability of bioinformatics methods to find useful correlations between gene sequences and biotopes. This motivates the use of a large set of categories organized in a hierarchical structure. Moreover, [14] has shown that the lexical information contained in ontologies can make the task easier.

OntoBiotope is an ontology of microorganism habitats [15]. Its modeling principle and its lexicon reflect the usual biotope classification used by biologists to describe microorganism isolation sites (*e.g*. GenBank, GOLD, EnvO) [16-18]. OntoBiotope is developed and maintained by the *Meta-omics of Microbial Ecosystems *(MEM) network in which 30 microbiologists from INRA (French National Institute for Agricultural Research) from all fields of applied microbiology participate. The relevance of OntoBiotope terms has been evaluated through the *PubMedBiotope *semantic search engine [19]. It identifies and categorizes biotopes in a collection of 600 000 PubMed abstracts by applying the ToMap method (*Text to Ontology Mapping*) [20] to the OntoBiotope ontology. This suggests that the ontology is fully appropriate as a new fine-grained categorization plan for the BB'13 task.

The BB'11 corpus is a collection of encyclopedia-like web pages. They are comprehensible by non-biologists and they share many linguistic characteristics in common with scientific articles. The limited size of encyclopedic webpages, compared to full research articles, is appropriate for a first attempt at a novel task, while preserving the generalization of trained IE systems.

## Methods

This section describes the corpora features, the event representation and the evaluation metrics for the two tasks. More details and examples can be found on the task website [21] and the ACL BioNLP Shared Task articles that are devoted to the two tasks [22,23].

### Gene Regulation Network

#### Biological and molecular representation in GRN

**Figure 1:**
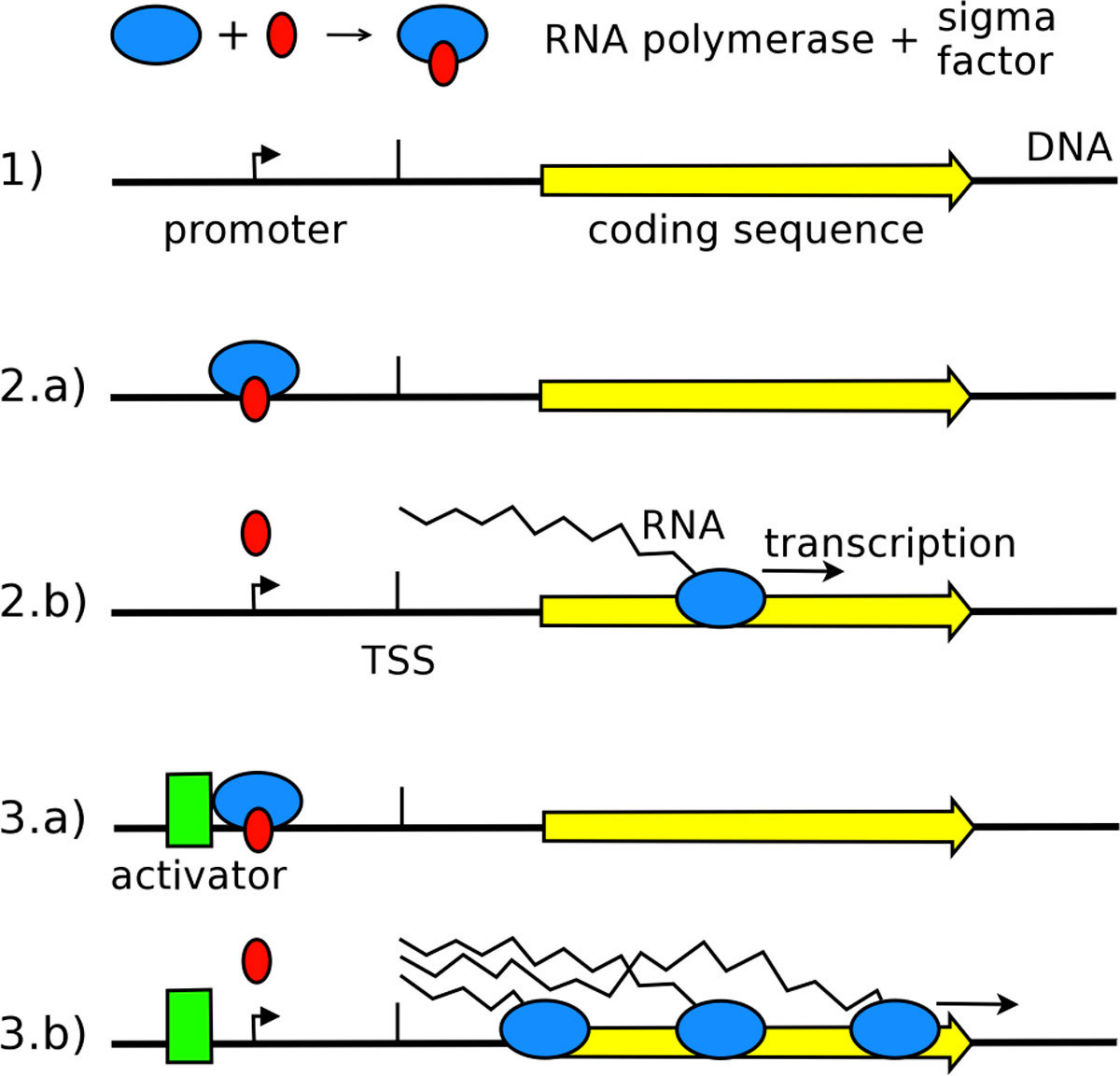
**Sketching out regulatory bacterial gene transcription molecular mechanisms**. (1) The RNA polymerase must bind to a transcription factor called sigma factor, to be able to transcribe DNA to RNA. (2.a) The sigma factor specifically recognizes a transcription promoter DNA sequence motif, upstream part of the gene, and drives the RNA polymerase to it. (2.b) DNA is copied into RNA from the Transcriptional Start Site (TSS), while the sigma factor is released and available for another RNA polymerase. (3.a) A transcriptional regulator binds to a specific motif around the promoter site, and in this example (3.b) activates transcription.

The goal of the GRN task is the extraction of a regulation network from text. The network is represented by a directed graph where the nodes are the genes and the arcs represent the interactions between them. Biological studies qualify the kind of interaction between biological entities according to the *effect *of the agent on the target, or to the *mechanism *by which the agent regulates the target. We defined six interaction types for the GRN regulation network representing the whole range of effect and mechanism regulation types [22]. The effect can be either *Activation *(positive regulation), *Inhibition *(negative regulation), or *Requirement *(the agent is necessary). Additionally, regulations can either be direct, which means that the agent protein physically interacts with the target gene, or indirect, which means that the regulation may be the result of a cascade of interactions. Direct regulations are particularly informative; the literature describes direct regulations at the molecular mechanism level as either physical *Binding *of a protein to DNA, or as the effect of a protein on gene *Transcription *as represented in Figure 1.

Molecular mechanisms are frequently detailed in the literature on bacteria and are very useful to determine the nature of the regulation. Moreover, the events involve not only proteins and genes, but also parts of them, families, complexes, or even cascades of nested events.

**Figure 2:**
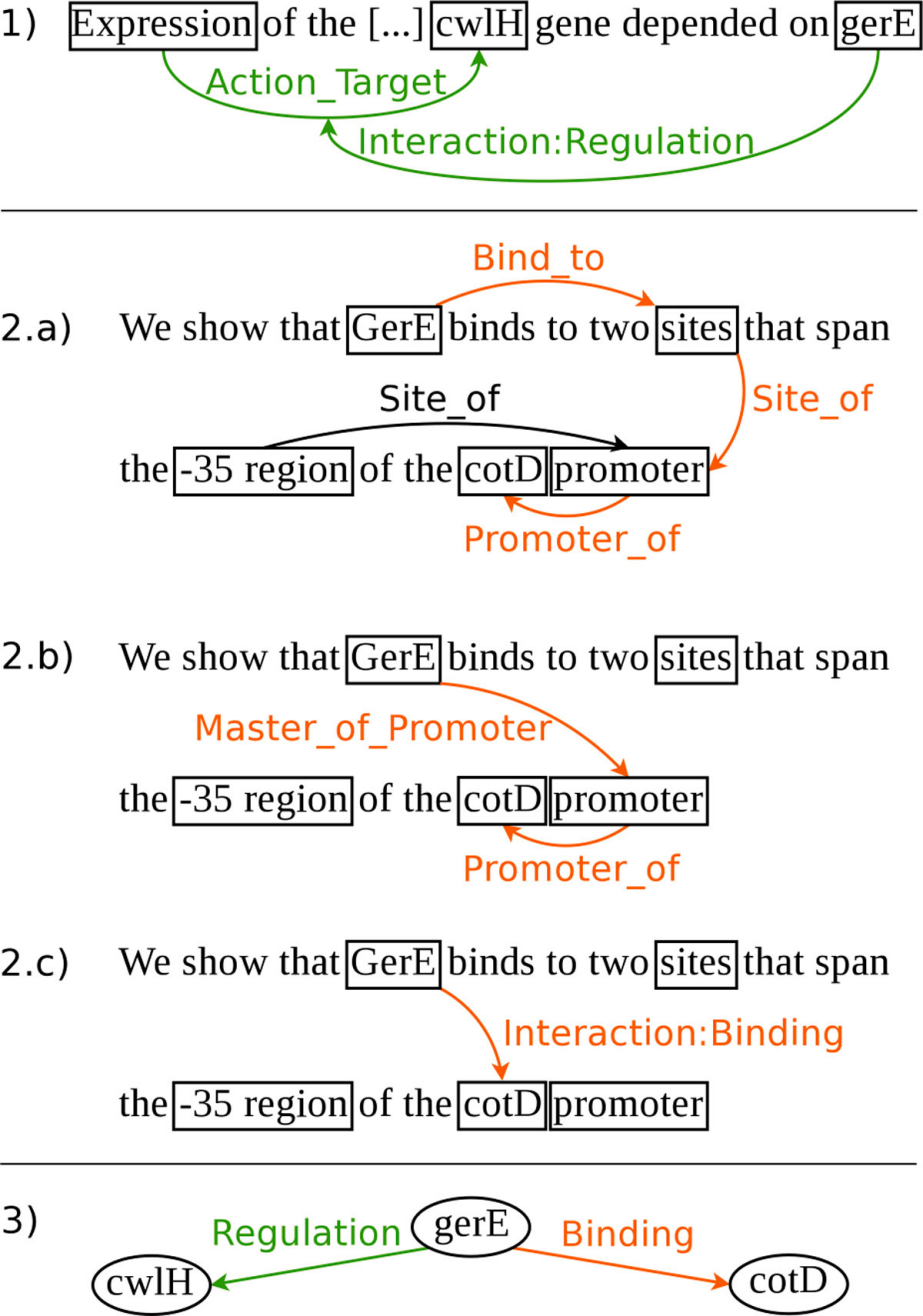
**From molecular mechanisms to biological interaction**. 1) the network arc between gerE and cwlH in Ex. 3 is inferred from the Interaction:Regulation event. Since the interaction target is an event, the target is reduced to its participant (cwlH); 2) a) Bind_to and Site_of represent low-level biological phenomena from which we can deduce the Master_of_promoter relation of 2) b); c) the Interaction:Binding relation can be deduced from Master_of_Promoter and Promoter_of; 3) the combined interactions from examples 1) and 2) produce the network with 3 edges and two arcs.

The six types of gene interactions are thus not sufficient to represent the whole complexity of interaction descriptions: a more comprehensive annotation model is necessary. It should allow the biologists to annotate the corpus by ensuring that entities, relations and events map easily to text elements and have an unequivocal biological interpretation. It must also be flexible enough to contend with linguistic phenomena like ellipsis and metonymy. The GRN annotation model meets these requirements. It accurately represents biological concepts and phenomena and is suited for text annotations, from the most generic indirect interactions (*e.g*. "X inhibits the expression of Y"), to the most detailed descriptions of physical interactions. The text annotation model comprises two levels: the biological level and the molecular level. The biological level includes (1) the same interaction type events as the regulation network, (2) transcription events (*Transcription by *and *Transcription from*) and (3) regulon membership events (*Member/Master of Regulon*). The interaction type events will be denoted by Interaction:*type *in the following, as in Interaction:Regulation. Network inference rules automatically and directly infer network arcs and nodes from text annotations. In cases where the interaction arguments are not genes or proteins, but nested events, the events are reduced to the participating genes. Example 1 of Figure 2 illustrates a nested event and Example 3 shows the resulting Regulation arc.

The molecular level of the annotation model represents the role of the promoter of the regulated gene and the binding of the protein on the promoter as illustrated by Example 2 of Figure 2. The model defines a *Master of Promoter *event that relates the binding protein to the promoter and more generally the *Bind to *event relates the binding protein to any site. The model also defines a *Promoter of *event that relates the promoter to its gene and more generally the *Site of *event relates any binding site to its DNA region (gene or promoter).

Inference rules derive the Interaction arcs of the network and their types from these molecular low-level events. Inference is done in two steps, (i) inference of biological annotations from the molecular annotations, and (ii) inference of the network arcs from the biological annotations. Example 2 of Figure 2 illustrates the inference of molecular to biological annotations. Example 3 shows the result of the inference of the network arc *Interaction: Binding*.

[22] formally specifies the annotation model and the inference rules that produce the regulation network from the text annotations. The specifications were made available on the GRN BioNLP-ST 13 webpage, as well as a tool for checking the predicted events against the annotation model and for inferring the network from these predictions.

### Information extraction challenges in the GRN task

The GRN corpus has been designed by following the BioNLP annotation standard [3]. It was selected from Pubmed abstracts on *Bacillus subtilis *transcription. All together, the information represented by the corpus had to be sufficient to build a regulation network centered on the sporulation of *Bs*.

The annotation model is based on the *Bacteria Genic Interaction *(BI) proposed in BioNLP-ST 2011. The manual annotation of the whole GRN corpus confirmed that it captures all of the descriptions of genic interactions without ambiguity. The regulation network that has been inferred from the annotations has been checked against state of the art knowledge [24,25] with a focus on the sporulation of *Bacillus subtilis *[26]. Its formal validity was checked by applying the inference rules to the corpus annotations.

Unlike most task corpora, the GRN corpus is a set of sentences isolated from PubMed abstracts. This is done for two reasons. Isolated sentences provide all the regulation network information. The prediction of the correct relations among the entities in the sentences is challenging as previously demonstrated by the LLL and *Bacteria Genic Interaction *(BI) tasks. This challenge is the result of a high number of entities (almost 5 per sentence on average), their diversity (11 types) and the diversity of the events (15). The sentences are provided with the gold entities (genes, proteins, promoters, *etc*.) and their text span, allowing the participant methods to focus on relation extraction.

The GRN corpus was split into the training, development and test sets, ensuring that the distribution of event types and entities in the training and development sets was representative of the test set. The molecular level annotations account for 60% of the annotations, and the biological level interactions for 40%. At the biological level, *Transcription *and *Regulation *interactions combined account for half of the interactions.

The small number of arcs in the network compared to the number of events is due to two factors (Table 1). First, some regulations are repeated in the text and represented by a single arc in the network. Second, some of the network regulations are inferred from several molecular events.

**Table 1:** Figures of the GRN corpus.

Sentences	201
Words	4 936
Molecular events	495
Biological Interactions	334
Events	819
Entities	917

Network nodes	133
Network edges	242

### Prediction evaluation metrics

The evaluation must assess the quality of the predicted gene regulation network with regards to the knowledge contained in the corpus. The reference network has been inferred from the manual corpus annotations using the inference rules detailed above. Therefore, the evaluation compares the predicted networks to the reference network.

The evaluation accepts predicted networks in two possible formats. In the first, the submission includes the prediction of text-bound events from which the predicted network is inferred. Alternatively, regulation network submissions without any text annotations are also valid. These formats allows for a greater diversity of prediction methods. They may or may not use the low-level annotations and the inference rules. They can even make use of information from external sources in order to build a network prediction.

**Figure 3:**
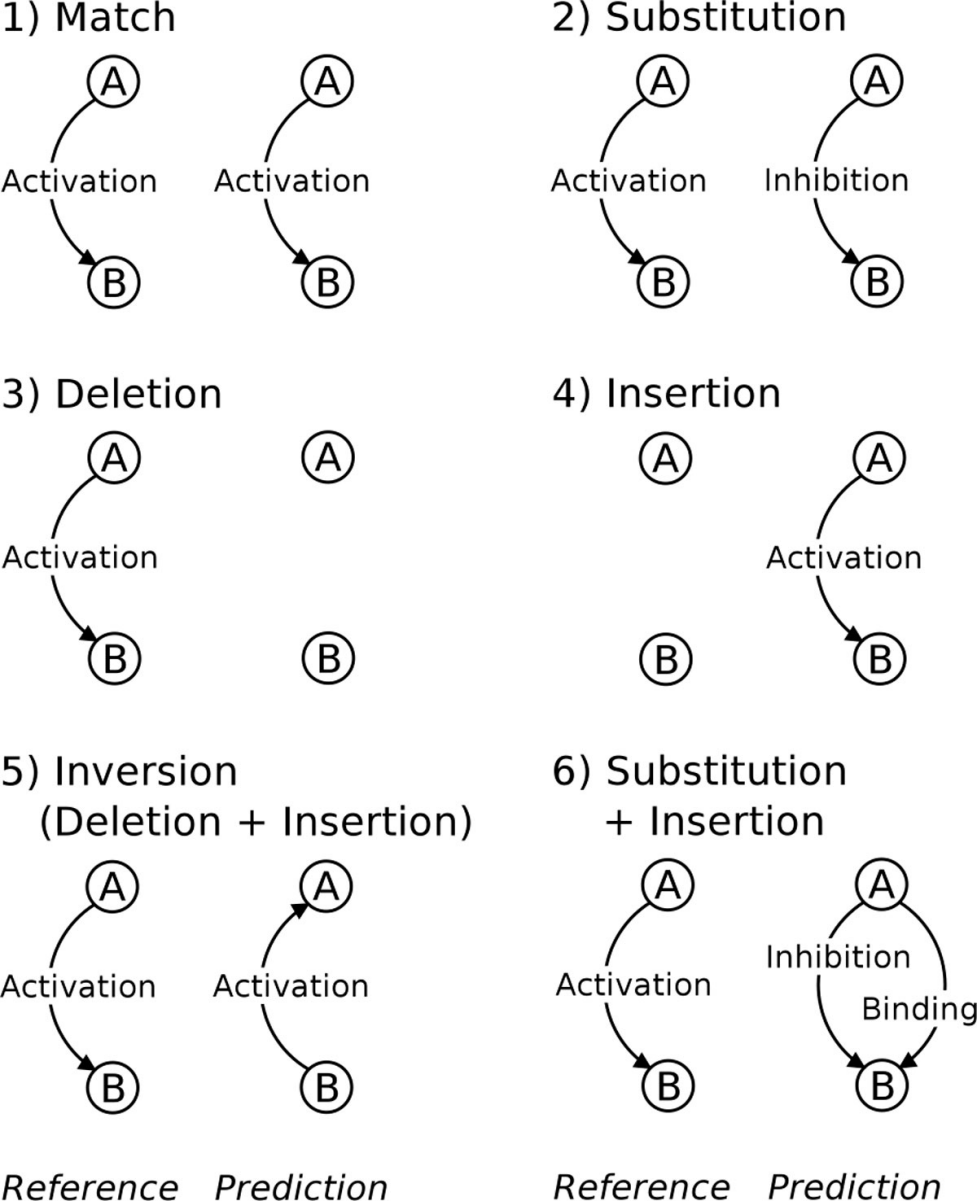
**Typology of errors in the GRN network**.

The evaluation algorithm compares two directed labeled graphs, the first one representing the reference network and the second representing the predicted network. Both graphs have the same set of edges and the comparison can rely exclusively on the comparison of arcs because the annotated corpus accounts for all of the gene mentions, along with their normalizations. Figure 3 illustrates the different comparison cases for two networks represented by a hypothetical 2-edge graph.

Two kinds of errors are noteworthy. The *inversion *error reverses the direction of an interaction by confusing the agent and the target roles. It is quite detrimental for the design of a systems biology model because the inversion can cause negative side effects that are costly to recover. The *substitution *error occurs when an arc is correctly predicted, but its label is incorrect. From the target application point of view, the cost of *a posteriori *recovery of a substitution error is equivalent to the correction of a false positive (FP) or a false negative (FN). However, an evaluation framework without substitution errors counts these errors twice: as a false negative and as a false positive. In the case of the F-score, calculated on the basis of FP and FN errors exclusively, both recall and precision account for each substitution. Since the F-score is the harmonic mean of recall and precision, it penalizes the substitution twice, overestimating the deviation from the reference. These standard metrics are therefore inadequate for GRN. Instead, we use the Slot Error Rate (SER) as proposed by [27]. The SER is related to the Levenshtein distance and to the Word Error Rate (WER) that is widely used in speech recognition evaluations.

The SER is defined as:

$$SER=\frac{S+D+I}{N}$$

where *S *is the number of substitutions (mismatches), *D *the number of deletions (false negatives), *I *the number of insertions (false positives) and *N *the number of items (arcs) in the reference. The SER indicates the proportion of errors in a prediction in comparison to the reference. The lower the SER, the better the prediction. A SER equal to zero means that the prediction is perfect. However the SER is unbound since the number of insertions is unbound. By design, the SER requires an analysis that isolates the substitution errors and allots them the same weight as insertions and deletions.

The GRN evaluation algorithm handles the arc prediction errors for all of the pairs of genes mentioned in the test set as shown in Figure 3. The number of errors for the whole graph is therefore the sum of errors for each individual pair.

Although the SER has already been used to evaluate NER and IE tasks [28], it is still an unconventional measure. The interpretation of SER as an absolute figure is difficult due to a lack of familiarity. For convenience we also compute the recall, precision and F-score:

$$Recall=\frac{M}{N}\;\;\;Precision=\frac{M}{P}$$

where *M *is the number of correct matches and *P *the number of predicted arcs. However, the F-score should be not used as an absolute indicator of the performance of a prediction. The ranking of the participant systems according to either the SER or F-score may show discrepancies depending on the proportion of substitutions amongst errors. If two predictions exhibit comparable SER scores, one may have a significantly lower F-score if it contains more substitutions than the other.

The SER measure used in GRN is thus aware of the recovery cost with respect to the application need. Its computation breaks down the prediction errors in a way that meets the expectations of the target biology community.

The predicted gene regulation networks are used for different purposes, whose requirements vary in terms of accuracy. We distinguished two main usages for which we conducted two complementary alternative evaluations. First, Systems Biology applications require very high quality and manually curated models. The predicted network cannot be used directly as is, rather it facilitates bibliographic searches by pointing out relevant sections in the literature. In this context, the important information is contained in the topology of the network, compared to the exact categorization of the regulations. We therefore specify the *shape *evaluation by removing the regulation types, both in the reference and the predicted networks. Multiple arcs between gene pairs are reduced to one. Thus, in the *shape *evaluation, there is either no arc or there is one arc between two genes. Furthermore, there are no longer any substitution errors. There is no objection to the use of F-score for the *shape *evaluation and the F-score ranking is the same as the SER ranking.

The second alternate evaluation focuses on gene regulations of *effect *types. Effect regulations indicate the functional influence of an agent on a target gene. As for pathway models, the main expectation for a regulation network is a graph with arcs labeled with effect types, *i.e*. the generic Regulation type and the Activation, Inhibition, Requirement types. We thus designed the *effect *evaluation framework by removing all mechanism regulation types (*i.e*. Binding and Transcription). If there is a single mechanism type arc between two genes, then this arc is relabeled as a generic Regulation. If there are one or two effect type arcs along with a mechanism arc, then the mechanism arc is removed (it is in fact relabeled as a Regulation arc and it becomes redundant with the effect arcs). Since there are different types of effects, substitution errors may occur and therefore the F-score remains inaccurate and SER is preferred.

These three evaluation settings: the official shared task evaluation, the network shape evaluation and the evaluation of effect regulations, provide useful means to assess the participant method results from several different perspectives.

### Bacteria Biotopes

The BB task aims to extract text-bound entities and events. It involves three kinds of entities: bacteria, geographical places and other habitats. The last two are defined as bacteria biotopes. All entities have to be detected, meaning that their character position in the document must be predicted. The entity spans may be discontinuous. This extension of BB'11 allows us to represent entity strings that are segmented into non contiguous parts. For example, the *animal intestine *entity in the text *animal and human intestine *is discontinuous and overlaps with the *human intestine *entity. Only habitats need to be categorized. Habitat categorization is characterized as the assignment of relevant concepts of the OntoBiotope ontology. The version of OntoBiotope that is used for the BB task defines 1,756 concepts in a hierarchical structure. The deepest point of the ontology contains ten levels.

The two BB task events (*Localization *and *PartOf*) are binary events. Localization links bacteria entities to their biotope entities (geographical and habitats). Many habitat entities are physically embedded such as organs in hosts, or substances in containers. This information is particularly important in the case of hosts where the interaction of the bacteria with an organ strongly depends on the host itself. Therefore, the role of the PartOf event is to link sub-parts of living organisms that are bacterial hosts, to the living organisms themselves.

The detection and categorization of entities and the extraction of events among them are considered separately in two distinct sub-tasks. These two sub-tasks are combined in the form of a third sub-task. Compared to BB'11 where all of the goals were combined in a single task, for BB'13 the efficiency of IE methods can be evaluated with respect to the different goals.

- The novel goal of entity categorization with a large ontology deserved a specific sub-task. The goal of sub-task 1 is the detection and categorization of habitat entities.

- The goal of sub-task 2 is the extraction of the *Localization *and *PartOf *events between entities of the three types. The annotations of all candidate entities are provided to the participants. By separating this sub-task, the measure of the event extraction quality is independent of the measure of the entity extraction quality.

- Finally, the goal of the third sub-task is the complete task, including the detection of entities of their three types without their categorization and the extraction of the two types of events.

**Figure 4:**
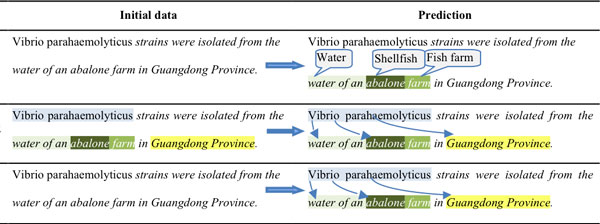
**Examples of the provided information and the expected prediction in BB'13**.

Figure 4 gives examples of the annotations provided to the participants and the expected predictions for the three sub-tasks.

### Corpus characteristics

Table 2 gives a summary of the distribution of entities and events in the BB'13 corpus. The BB corpus texts were selected from the collection of bacteria description web pages used in BB'11 [4]. The selection ensured that the distribution of the annotations and the linguistic phenomena are representative [23]. The analysis of the corpus peculiarities with respect to the task goals gives an insight into the potential difficulties for the information extraction methods.

**Table 2:** Figures of the Bacteria Biotope corpus annotation.

Document	131
Word	43,851
Bacteria	2,220
Geographical	288
Habitat	2,675
OntoBiotope cat.	2,097
*Total entities*	5,183
Localization	1,837

Part of Host	475
*Total events*	2,312

### Entities

The morphological similarity between ontology concepts and the entities to be tagged can be used to facilitate the categorization. We found that 60% of BB corpus habitat entities have forms different from the concept or synonym that they should be tagged with. A straightforward and naive strategy consists of a direct match of ontology habitat entries to the test text after lemmatization. It yields a high Slot Error Rate (SER) of 0.74, which is a low baseline. The participant scores range from 0.46 to 0.66 SER, significantly better than the baseline. This shows that category assignment is a non trivial problem.

The repetition rate of the entity occurrences and of their categories is an important factor to take into account when designing the prediction method. A quarter of the habitat entities occur more than once in BB'13, which is a significant proportion. It represents half of the total number of habitat occurrences. Only a small number of repeated entities (112 occurrences) belong to several different ontology categories. Consequently, the propagation of the most likely category annotations of a given entity to all its occurrences is a first-line strategy.

Discontinuous entity annotations may also be difficult to detect automatically. Table 3 gives the typology of discontinuous annotations with their distribution in the corpus. In most cases, discontinuous annotations occur in coordinations where the head (*intestine *in *animal and human intestine*) or the argument is shared by the members of the coordination. Range structures such as in *regions with tropical to sub-tropical climate *are also frequent in bacteria biotope documents. They are mostly used to describe physico-chemical conditions. A small rate of entity annotations are discontinuous (96, or 1.8%), and most of them are habitats. Processing discontinuous entities should therefore have a low impact on the information extraction method results.

**Table 3:** Distribution of discontinuous annotations in BB'13 corpus.

Linguistic structure	Frequency
and	68
or	11
enumeration	6
range	5
combination of insertion and coordination	1
tmesis (insertion)	6
Total	97

### Events

The PartOf and Localization events account for one quarter and three quarters of the events, respectively. The frequency of PartOf events in the corpus is far from negligible. The scores obtained by BB'11 participant methods on the two event types were similar, with a slightly lower score for PartOf.

Events whose arguments are local to the sentence (intra-sentence events) are easier to extract. Cross-sentence extraction remains challenging because it may require anaphora resolution. In the BB'13 corpus, half of the Localization event arguments (54%) occur in the same sentence, while the rate is higher (63%) for the PartOf events (Table 4). The methods that use a representation of the examples that is based on individual sentences, such as syntactic dependency paths between the event arguments, cannot identify all events without an additional step to deal with inter-sentence events. It should be noted that almost all of the arguments for the two events are found within the same paragraph, as shown in the third column of Table 4. This significantly restricts the range of argument candidates, in particular the bacteria argument of the Localization event.

**Table 4:** Distribution of the event arguments in the text.

	% intra sentence events	% intra paragraph events
Localization	0.54	0.94
PartOf	0.63	0.98
All events	0.56	0.95

Human annotators frequently cannot choose a single bacterium name occurrence as the valid argument of a Localization event, to the exclusion of all other occurrences of the name. In this case, the annotator attaches an equivalence set of relevant bacteria occurrences to the event. The prediction of any of the members of the equivalence set is considered equal, and therefore evaluated as valid.

A significant portion of the entities are not involved in any events. This affects more than half of the bacteria and one third of the habitats and geographical places (Table 5). This is unusually high compared to the other BioNLP tasks and may require the development of an adequate strategy.

**Table 5:** Rate of candidate arguments that belong to an event (Localization or PartOf).

**Argument Type**	**Habitat**	**Bacteria**	**Geographical**	**Entity**
Percentage of entities involved in events	63%	44%	65%	55%

### Corpus preparation

The preparation of the BB'13 corpus was completed following a three-step annotation process, for which we used the AlvisAE Annotation Editor [29]. First, the AlvisNLP pipeline automatically pre-annotated the entities and their categories to speed-up the manual annotation process. Next, eight biologists and computer scientists performed a double-blind manual annotation after a training period. They followed detailed guidelines that limited the number of different interpretations of the task goals. Finally, the conflict resolution phase resulted in the final annotation. This final phase was done using the AlvisAE conflict detection tool. The annotators also used a Forum and a Wiki to debate guidelines interpretations and to record their decisions. The guidelines were revised accordingly. A revision tracking tool identified and displayed the annotations that should be checked because they were potentially affected by subsequent revisions. The annotators achieved a consensus annotation by debating the conflicts.

**Figure 5:**
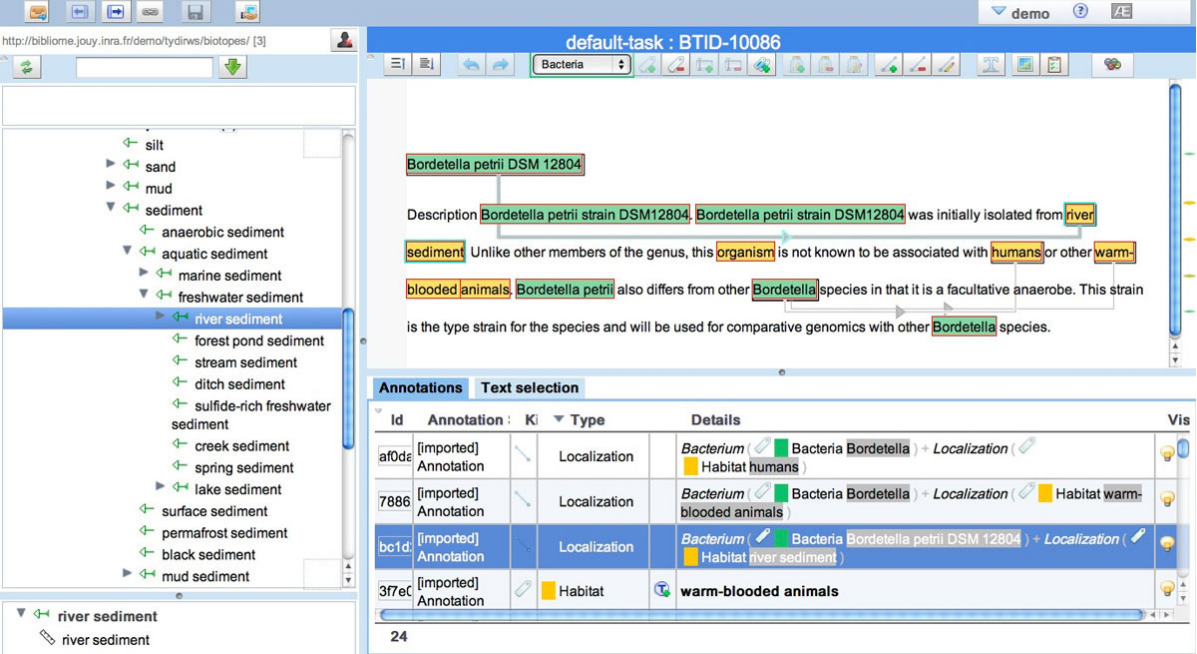
**OntoBiotope category assignment with AlvisAE annotation editor**.

Compared to the preparation of the BB'11 corpus, the annotation was facilitated in two ways: the use of the AlvisAE editor and the automatic pre-annotations. Using AlvisAE the manual annotation of events between entities, equivalence sets and entity categories is completed using simple clicks and drag-and-drops. The graphical display of the annotations makes validations and revisions easier, while limiting the risks of errors. The manual detection and categorization of entities is a time-consuming task, due to the high number of categories and entities to be tagged. AlvisAE supports the manual association of relevant OntoBiotope concepts to habitat entities by synchronizing the text annotation text and by displaying the ontology, as shown in Figure 5.

The automatic pre-annotation detects habitat entities and predict their categories. It had a strong impact on the manual annotation effort. It is supported by AlvisNLP. It is done by the method described in [14]. It consists of the terminological analysis of the corpus using the BioYaTeA term extractor [30] that detects candidate entities. Next, terms are tagged using the ToMap method that assigns the categories [20]. ToMap is based on the phrase similarity syntactic analysis principle, such as used in MetaMap [31]. However, it is applicable to all types of termino-ontologies and corpora in French and English. In order to measure the quality of the pre-annotation and estimate the gain in time, we evaluated the pre-annotation to the reference corpus using the BB'13 task metrics. Table 6 gives the scores obtained on the test corpus of sub-task 1 for the three pre-annotation steps: habitat entity detection, habitat entity categorization and the combination of detection and categorization. The categorization score is obtained using the boundaries of the reference entities. This score is of importance when measuring the impact of detection errors on further categorizations.

**Table 6:** Automatic pre-annotation scores by AlvisNLP.

	SER	Recall	Precision	F1
Detection & categorization	0.44	0.58	0.78	0.66
Entity detection	0.37	0.64	0.86	0.74
Entity categorization	0.34	0.67	0.90	0.77
Categorization with reference entity	NA	0.90	0.90	0.90

The ToMap categorization method is very efficient (90% F-measure) when applied to the reference entities. The F-measure significantly decreases by 24 points when ToMap is applied to predicted entities (F-measure = 0.66). A detailed analysis of the errors showed that the entity detection errors are in general deletion errors (429), rather than insertion errors (31) or incorrect boundaries. In other words, the BioYaTeA term analysis method is accurate for the prediction of entity boundaries. On the other hand, the ToMap method filters out too many correct entities when assigning categories. Conversely only a few of the incorrect entities are preserved and ToMap generally assigns the correct category to the remaining entities. In terms of time gain for the annotators, the pre-annotation method significantly facilitates the entity categorization, which was the main issue. On the other hand, the missed entities had to be manually identified by the annotators.

### Prediction evaluation metrics

The BB'11 metrics were used when possible for the sake of comparison of the BB'13 results to previous ones. The novelty of sub-task 1 required the design of new suitable metrics.

### Measure for entity detection and categorization

The goal of sub-task 1 is the prediction of habitat entities in text and their categorization. The recall and precision measures could be defined as usual,

$$Recall=\frac{S}{N}\;\;\;Precision\frac{S}{P}$$

where *N *is the number of reference entities and *P *the number of predicted entities. The prediction of imprecise entity boundaries and approximate categories should be counted as partial errors and not as full errors since an approximation of the biotope reference is better than no prediction at all. *S *represents the sum of the similarities *S *between reference entities and their corresponding partial matching entity. For the same reasons that were given above for GRN, the SER measure is more appropriate here than the F-measure since it overestimates the partial match errors.

In the SER formula, Insertions represent false positives *i.e*. predicted entities that do not overlap with any reference entity. Deletions represent false negatives, *i.e*. reference entities that do not overlap with any predicted entity. Substitutions are inversely proportional to the similarity between the predictions and the references that partially overlap.

The similarity between the predicted entity and the reference entity depends on two criteria: the similarity of their entity boundaries *S_e _*and the similarity of their categories *S_c_*.

In BB'11, the similarity *S_e _*of entity pairs was measured as the ratio between the size of the overlapping text segments and the size of the two merged text segments. *S_e _*is equal to 1 (maximum) if the two entities are equal and tends to be zero for barely overlapping segments. Formally, it is a variant of the Jaccard index applied to segments. The analysis of the BB'11 participant results demonstrated its significance [4]. We extended this measure to take into account the discontinuity of entity spans.

In the same way as for entity boundaries, approximate predictions of the entity categories should not be counted as full errors. Moreover, the evaluation should favor ancestor predictions over sibling predictions, since the prediction of an overly general category remains correct, even though it is less precise. Additionally, the prediction information wealth should decrease faster than the number of nodes on the path from the prediction to the reference. The Wang semantic similarity fits these requirements [32]. It has been successfully applied in previous work to compute semantic distances in ontologies [33].

The similarity *S_sub-task1 _*between the prediction and the reference for sub-task 1 is defined as the product of the entity similarity and the category similarity, yielding a measure between zero and 1,

$$S_{sub-task1}=S_{c}.S_{e}$$

The SER substitution factor is simply the opposite of the similarity *S_sub-task1 _*and is defined as:

$$S=1-S_{sub-task1}$$

### Measure for event prediction

The evaluation of sub-tasks 2 and 3 results measures the quality of the event predictions. Additionally, the evaluation of sub-task 3 measures the quality of the arguments of the correct events. These goals can be formalized as a categorization problem of all the pairs of entities for which recall, precision and F_1 _measures are appropriate. We then used the same setting as for BB'11. The accuracy of the biotope argument is measured by *S_e _*as in sub-task 1, whereas the accuracy of the bacteria argument is measured using a strict equality.

### Entity pairing

**Figure 6:**
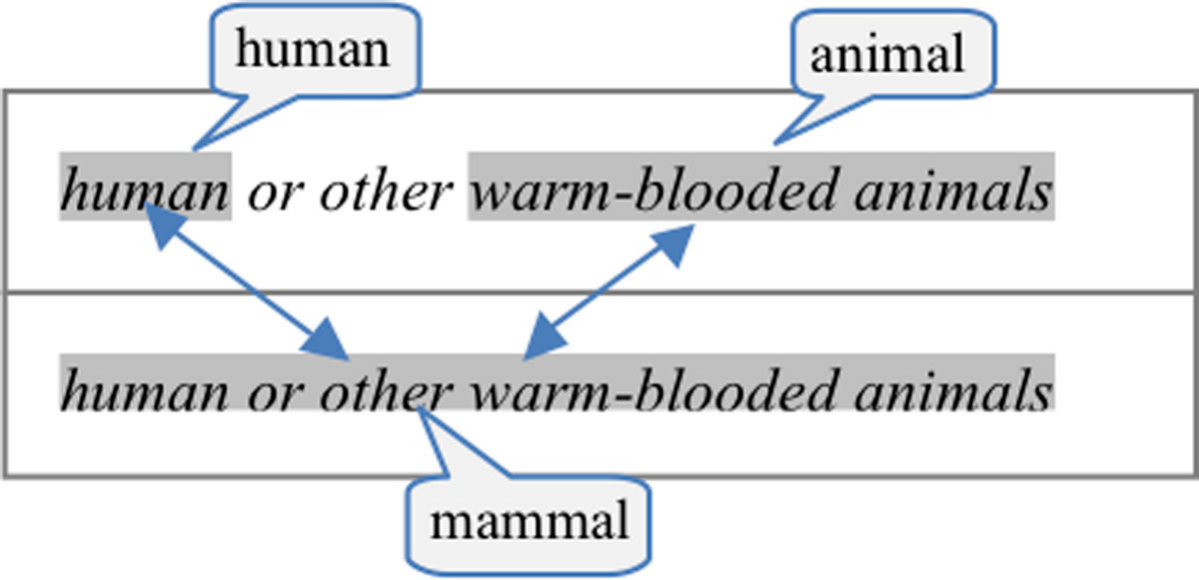
**Example of two possible matches between the reference and the prediction**.

The scores defined above assume that each predicted entity matches a single reference entity and *vice versa*. Given the approximation of boundaries and categories, the possible matching between prediction and reference is not unique as illustrated by Figure 6. We defined the criteria for selecting the pairing as the one that maximizes the participant prediction score.

## Bacteria Track results

Five teams participated in BB, five teams in GRN and two teams participated in both. We analyzed the relevance of the corpus and evaluation metrics, the method strengths and their relevance for biological applications with respect to the results. The comparison of the 2013 results to the previous series that took place two years ago shows the evolution of community methods on challenging information extraction tasks.

### Gene Regulation Network

Table 7 shows the evaluation results and the characteristics of each GRN submission. The best prediction scored an SER of 0.73 (*University of Ljubljana*). The GRN task attracted submissions that use diverse prediction methods. Two participants, *EVEX *[34] and *K.U.Leuven *[35], directly submitted a network graph (Table 7). The remaining three teams submitted text-bound events that were evaluated using the inferred network. One of them, *IRISA *[36], only submitted the high level text-bound Interaction events. The other two teams, *University of Ljubljana *[37] and *TEES-2.1 *[38], submitted both Interactions and low-level events.

**Table 7:** Official scores of the GRN task.

Participant	Submission	ML algorithm	SER	Recall	Precision	Shape SER	Effect SER
U. of Ljubljana	Interaction +low-level	LC-CRF	0.73	0.34	0.68	0.60	0.74
K.U.Leuven	Network	SVM	0.83	0.23	0.50	0.64	0.83
TEES-2.1	Interaction + low-level	SVM	0.86	0.23	0.54	0.74	0.84
IRISA	Interaction	kNN	0.91	0.41	0.40	0.51	0.87
EVEX	Network	-	0.92	0.13	0.44	0.79	0.91

The distinction between the three types of errors (deletion, insertion and substitution) allows us to better qualify the strengths and weaknesses of the predictions. Except for IRISA, all predictions have roughly the same error profile: a high number of missed arcs and a relatively low number of substitutions and incorrect insertions of arcs. This is reflected by the low recall that ranges from 13% to 34%, while the precision ranges from 44% to 68%.

The *IRISA *submission shows a much more balanced profile with nearly the same number of substitutions, deletions and insertions. This submission is also bolder since its network contains more than twice as many arcs (91) than the others (the second by *University of Ljubljana *has 44 arcs).

The results of the *shape *evaluation (without regulation types) are much more optimistic, even though the relative ranking of submissions remains unchanged with the noticeable exception of *IRISA *(from fourth to first) with a 75% F-score and an impressive gain of 0.40 SER. Regardless of their ranking, all predictions yield a high precision score: the highest is 88% for the *University of Ljubljana *team and the lowest is 74% for *IRISA*, whose main strength is the recall compared to the others.

We conclude that systems are better at predicting regulations, but less accurate at typing them. The *TEES-2.1, EVEX *and *University of Ljubljana *submissions show the smallest SER gains (+0.12, +0.12 and +0.13 respectively) suggesting that these systems are slightly more accurate in choosing regulation types.

In the *effect *evaluation (without mechanism types, Binding and Transcription), the ranking of submissions remains unchanged with a nominal SER change for all. We conclude that the prediction of mechanism labels is quite accurate for all systems and that the most challenging aspect of the GRN task is the determination of effect labels (Activation, Inhibition, and Requirement), which is most important for biological applications.

### Bacteria Biotope

Five teams submitted ten predictions to the three BB sub-tasks (Table 8) [39-41,38,36]. Provisional results were provided following the submissions; we present in this paper the definitive results. The participant teams had different performances with respect to the three sub-tasks that require different skills.

**Table 8:** Participation to the bacteria biotope task.

	LIMSI	LIPN	TEES 2.1	IRISA	Boun
Sub task 1	✓	✓		✓	✓
Sub task 2	✓		✓	✓	✓
Sub task 3	✓		✓		

### Habitat detection and categorization (sub-task 1)

Our analysis of the sub-task 1 results focuses on how the detection of entities and their categorization interact. We are also interested in the way in which the task metrics can help evaluate the method relevance for real-life applications.

Table 9 displays the prediction results of the five participants. Column a. gives the official results. In order to better evaluate the strengths and weaknesses of the methods with respect to the detection and categorization of entities, we computed alternative evaluation measures with relaxed constraints shown in columns b and c. Column b provides the measures for the entity prediction quality, without taking into account the categorization prediction at all. Column c gives the measures of the categorization quality without taking into account the quality of the entity boundary prediction. The methods obtain good results with over 70% F_1 _measure for the best results. The scores are significantly higher (10 points in average) than the official combined results of column a. This confirms that entity detection errors affect the categorization quality.

**Table 9:** Sub-task 1 results in BB'13.

	Official results (a)		Habitat Detection (b)		Category assignment with relaxed habitat boundaries (c)	
**Participant**	**SER**	**F_1_**	**SER**	**F_1_**	**SER**	**F_1_**

LIPN	0.661	0.608	0.629	0.639	0.550	0.718
Boun	0.676	0.595	0.617	0.653	0.554	0.715
LIMSI	0.678	0.444	0.467	0.714	0.637	0.496
IRISA	0.932	0.574	0.895	0.603	0.814	0.668

The new categorization challenge has been successfully completed despite its novelty. As highlighted in the Background section, there is a strong need for methods capable of normalizing biotope mentions, without necessarily extracting the exact entity text. These good results on sub-task 1 are thus very promising with respect to the application needs of the bioinformatics domain.

The methods also obtain slightly lower, but overall good results on the detection of entities, except for *LIMSI *[39]. The analysis of the detection results shows that the method errors are mainly insertion errors (false positives) rather than substitutions (wrong boundaries) and deletions (false negatives). This is confirmed by the entity detection results in Table 10 that are obtained by fully relaxing the boundary constraints. More precisely, this means that an entity prediction is counted as a true positive, if there is at least one character in common with the reference. Boundary relaxation significantly increases the detection results (Table 10). The differences are figured in the parentheses. All methods yield F_1_-measure scores over 70%. The insertion and deletion measures stay unchanged. It is worth noting that the four F-measure results are close although they were obtained using different strategies that favor recall (*IRISA*, 91%) or high precision (*LIMSI*, 92%). This confirms that an overall improvement can be expected with more accurate boundary predictions.

**Table 10:** Sub-task 1 entity detection results, with relaxed boundaries.

	SER	Recall	Precision	F1
LIMSI	0.308 *(0.370)*	0.716	0.920	0.819 *(0.375)*
Boun	0.479 *(0.197)*	0.824	0.804	0.814 *(0.219)*
LIPN	0.487 *(0.174)*	0.803	0.803	0.803 *(0.195)*
IRISA	0.775 *(0.157)*	0.909	0.601	0.724 *(0.150)*

However, there is a need in bioinformatics for methods capable of detecting biotope mentions without high boundary accuracy. Fast reading of relevant scientific documents is a significant example of such an application. This result, in fact, meets the core of the application needs, which is promising for future developments.

### Event extraction (sub-task 2)

Table 11 gives the results obtained by the participant methods on sub-task 2 where the entities were provided and the Localization and PartOf events had to be predicted. PartOf event predictions are significantly worse than Localization event predictions as opposed to BB'11. The discrepancy does not seem to be caused by the difficulty of PartOf extraction, but is rather suspected to be related to the participant methods, no general conclusion should be drawn. Higher precision than recall, as displayed in Table 11, shows that most of the methods have trouble generalizing from the training examples.

**Table 11:** Official results of sub-task 2 for BB'13 task.

Participant	Recall	Precision	F_1_	F_1 _PartOf	F_1 _Localization
TEES 2.1	0.28	0.82	0.42	0.22	0.49
IRISA	0.36	0.46	0.40	0.2	0.45
Boun	0.21	0.38	0.27	0.2	0.29
LIMSI	0.04	0.19	0.06	0.0	0.07

In the Methods section, the potential effect of the high rate of inter-sentence events on method efficiency was discussed. No significant increase in the scores was observed for the intra-sentence PartOf events. Table 12 shows the results obtained through the extraction of intra-sentence Localization events. The difference between the regular and the intra-sentence results greatly varies between methods. A 24 point improvement is observed for *TEES-2.1*. This method uses syntactic path features in order to describe intra-sentence events. This hypothesis is confirmed by the high recall increase (+31 points). By contrast, the rote learning method of *LIMSI *is not affected.

**Table 12:** Results of sub-task 2 measured on intra-sentence events.

Intra-sentence		Scores		Difference with the full task scores		
	**Recall**	**Precision**	**F_1_**	**Recall**	**Precision**	**F_1_**

TEES-2.1	0.51	0.82	0.63	+0.23	0	+0.21
IRISA	0.37	0.38	0.37	+0.01	-0.08	-0.03
Boun	0.19	0.29	0.23	-0.02	-0.09	-0.04
LIMSI	0.03	0.17	0.05	-0.01	-0.02	-0.01

**Intra-sentence (Localization)**	**Recall**	**Precision**	**F_1_**	**Recall**	**Precision**	**F_1_**

TEES-2.1	0.66	0.82	0.73	+0.31	0	+0.24
IRISA-TexMex	0.48	0.38	0.42	+0.04	-0.08	-0.03
Boun	0.18	0.27	0.22	-0.05	-0.11	-0.07
LIMSI	0.03	0.30	0.06	-0.01	0.01	-0.01

The methods of *IRISA *and *Boun *are slightly affected by the inter-sentence evaluation since their scores decrease by 3 and 7 points, respectively. Different reasons can be suspected. *IRISA *method is based on a word-based language model that does not use sentence boundaries. The method may be sensitive to the length of the argument context that is shorter and less discriminant on average in single sentences than in the general case. This explains the precision decrease for intra-sentence extraction (-8 points) that could be due to over-generalization. *Boun *method selects the first mention of bacteria in a given paragraph as the bacteria argument of any Localization event in the paragraph. The intra-sentence evaluation focuses on local events where this method strategy fails more frequently, which explains the decrease in precision (-11 points). The impact of intra-event extraction compared to the regular task is very different depending on the participant methods. Linguistic strategies such as anaphora resolution for linking entity references are critical for certain methods, such as syntactic dependency-based approaches, but potentially not useful for sequence-based learning.

### Entity detection and event extraction (sub-task 3)

Sub-task 3 combines the extraction of the events and the detection of the entities. The subtask entities include not only habitats, but also geographical places and bacteria. Table 13 gives the results of the two participant methods. The extraction of the entities significantly penalizes the results of *TEES 2.1 *compared to the results of sub-task 2 (-28), whilst *LIMSI *achieved similar results. The cause of the low scores is different depending on whether the method is better at entity detection (*LIMSI*) or event extraction (*TEES 2.1*). Table 14 shows the prediction quality of biotope entities (habitat and geographical), which is rather good (81 F-measure for the best).

**Table 13:** Scores on sub-task 3 of BB'13 Task.

Official scores				Scores with relaxed biotope boundaries			Scores with relaxed bacteria boundaries		
**Participant**	**Recall**	**Precison**	**F_1_**	**Recall**	**Precision**	**F_1_**	**Recall**	**Precision**	**F_1_**

TEES 2.1	0.12	0.18	0.14	0.41	0.61	0.49	0.28	0.52	0.36
LIMSI	0.04	0.12	0.06	0.09	0.82	0.15	0.07	0.71	0.10

**Table 14:** Scores on biotope detection in sub-task 3 of BB'13 Task.

Participant	SER	Recall	Precision	F1
LIMSI	0.32	0.68	1.00	0.81
TEES 2.1	0.50	0.57	0.76	0.65

The relaxation of the biotope boundaries (table 15) results in a greater improvement for the event extraction results of *TEES 2.1 *than *LIMSI*, which produces more false positives and negatives than substitutions. The relaxation of the strict boundary constraints for the bacteria also increases F_1 _for both methods as shown in table 13. The gain in precision is notably much higher for *TEES 2.1 *(+22 points) that often predicted fragmented bacteria names, such as for instance *D*. and *VCD115*, from *D. deserti VCD115*. The method of LIMSI also produces false negative predictions of bacteria. The use of a relevant dictionary of bacteria names such as the NCBI taxonomy should notably improve the global results [4].

**Table 15:** Scores on intra-sentence Localization extraction in sub-task 3 with relaxed entity boundaries.

Participant	Recall	Precision	F_1_
LIMSI	0.08	0.79	0.15
TEES 2.1	0.72	0.56	0.63

Not surprisingly, the best scores are achieved by both methods when both bacteria and biotope boundaries are relaxed and only intra-sentence event extraction is measured (table 15).

This detailed analysis of the evaluation measures thus provides valuable suggestions and ideas regarding the issues that need to be addressed. Entity boundary detection (especially for bacteria) and inter-sentence events seems to be the main hindrances. The first challenge could be easier to complete than the latter, given the high scores obtained in BB'11.

## Conclusions and Discussion

The Bacteria Track is motivated by the evolution of the biology field. The biology research domain is undergoing a shift in terms of the scale of experiments and their analysis. High throughput experiments provide comprehensive data over a large range of species. Whole cell models and cross-species metagenomics studies are conceivable provided experimental data is accurately linked with knowledge corpora contained in the literature. The goal of the Bacteria Track tasks is to demonstrate that the BioNLP community is well-grounded to accompany the progress of Microbiology research.

The GRN task targets biological processes and whole cell models, whereas BB targets ecological information for a large spectrum of bacteria species. The GRN and BB task definition and evaluation procedures are tailored to the biology knowledge modeling goals. The two corpora provide a benchmark for BioNLP IE systems that aim to measure their ability to build relevant knowledge bases. The results of participant systems on the Shared Task provide invaluable insight into their strengths and limits from which a number of conclusions can be drawn regarding the most promising research trends. For both tasks we have proposed the Slot Error Rate (SER) as a relevant evaluation measure. The SER has effectively allowed us to discriminate the performance of the submitted predictions. In particular, it contributed to a better characterization of the strengths of each submission by distinguishing three types of errors: Insertions (false positives), Deletions (false negatives) and Substitutions (mismatches).

### Gene Regulation Network task

The goal of the GRN task is to present systems biologists with a regulation network, rather than a set of text-bound events. Participant systems are able to produce results that biologists can immediately grasp and evaluate. Methods based on low-level events achieved using the three levels of annotations and the domain expert inference rules. The production of knowledge from text *and *domain rules is a new IE paradigm that differs from the usual BioNLP information extraction framework. The analysis of the submissions showed that participants predicted mainly the low-level events that were involved in the most formalized inference rules. The other low-level events were rarely predicted or not predicted at all. More focused and distinct processing for the extraction of elementary events and the design of the abstract regulation network should improve the quality of the results. In the future additional formalized inference rules should also allow systems to focus on the extraction of elementary events and improve the quality of inferred regulation networks.

The annotation model of the GRN corpus has characteristics in common with the GE and GRO corpus models [42,43] that also address molecular biology questions. A unified shared model for the tasks should make the generalization of the participant systems easier for all three tasks. The *EVEX *submission was a promising attempt towards this goal. Moreover, the GRO annotation model heavily relies on the Gene Ontology that could be used for the convergence of the three models.

### Bacteria Biotopes task

The results on the BB sub-task 1 (entity recognition and categorization) are very promising with respect to the novelty of the goal. The evaluation score combines boundary and categorization accuracy in a single measure. We have shown that incorrect boundaries negatively impact the categorization and are thus penalized twice. An even more application centered evaluation metrics might reduce the impact of boundary accuracy. The results on the BB sub-tasks 2 and 3 (event extraction with or without gold entities) are below the scores on similar extraction tasks that contain only a few event and entity types. After error analysis of the predictions, we indicated plausible means of improvement. In particular, relevant approaches for the prediction of bacteria names could be applied. The long distance events between bacteria and their biotopes deserve a specific treatment.

As was done after previous BioNLP shared tasks, the data, evaluation services and resources for the two tasks were made available. The test answers are not public in order to ensure that the comparison of future results remains possible.

## Competing interests

The authors declare that they have no competing interests.

## Authors' contributions

RB coordinated the task organization. RB and PB specified the GRN task and annotated the GRN corpus. RB, WG, CN, ZR, DV contributed to the specification and annotation of BB. RB, WG, CN achieved the corpus analysis for both tasks. All authors contributed, read and approved the manuscript.

## Declaration

The publication costs of this article were funded by the Institut National de la Recherche Agronomique (INRA, France).

This article has been published as part of *BMC Bioinformatics *Volume 16 Supplement 10, 2015: BioNLP Shared Task 2013: Part 1. The full contents of the supplement are available online at http://www.biomedcentral.com/bmcbioinformatics/supplements/16/S10.
